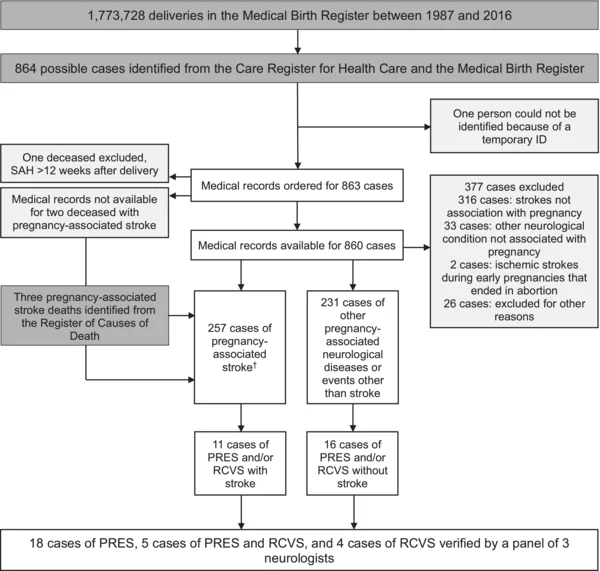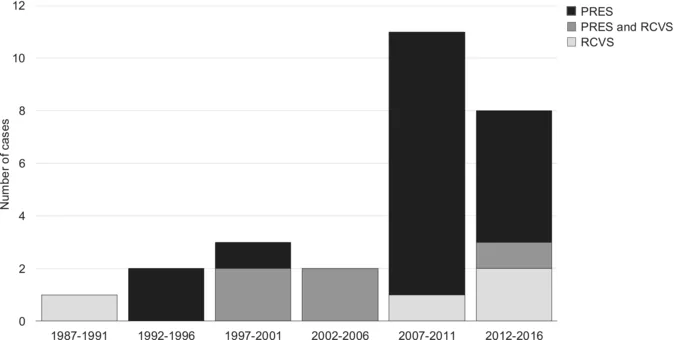# Correction to “A Population‐Based Study of Posterior Reversible Encephalopathy Syndrome and Reversible Cerebral Vasoconstriction Syndrome During Pregnancy and Puerperium”

**DOI:** 10.1111/ene.70748

**Published:** 2026-09-04

V. Teresa , V. Liisa , R. Kirsi , et al., “A Population‐Based Study of Posterior Reversible Encephalopathy Syndrome and Reversible Cerebral Vasoconstriction Syndrome During Pregnancy and Puerperium,” European Journal of Neurology 33, no. 5 (2026): e70615, 10.1111/ene.70615.42059197 PMC13129962

We have identified several errors in our article.

First, in the “Discussion” section (paragraph 2), we state: “Moreover, the incidence of PRES was 30 per 100,000 among eclamptic women in another study [12].” This statement is incorrect. The sentence should read: “Moreover, the incidence of eclampsia‐associated PRES was 0.03% (30 per 100,000 deliveries) in a previous study [12].”

Second, in Figure 1, the exclusion number “459” is incorrect and should be corrected to “377.” In addition, we suggest correcting minor grammatical errors ( e.g., replacing “associated to pregnancy” with “associated with pregnancy”).

Third, Figure 2 contains incorrectly labeled time periods. The period 1997–2002 should be revised to 1997–2001, and 2003–2006 should be revised to 2002–2006, reflecting the intended 5‐year intervals.

We apologize for these errors.